# Dysregulation of Neuronal Calcium Signaling via Store-Operated Channels in Huntington's Disease

**DOI:** 10.3389/fcell.2020.611735

**Published:** 2020-12-23

**Authors:** Magdalena Czeredys

**Affiliations:** Laboratory of Neurodegeneration, International Institute of Molecular and Cell Biology in Warsaw, Warsaw, Poland

**Keywords:** Huntington disease, huntingtin, Ca^2+^ signaling, neuronal store-operated Ca^2+^ channels, neuronal store-operated Ca^2+^ entry, medium spiny neurons, spines, huntingtin-associated protein 1 isoform A

## Abstract

Huntington's disease (HD) is a progressive neurodegenerative disorder that is characterized by motor, cognitive, and psychiatric problems. It is caused by a polyglutamine expansion in the huntingtin protein that leads to striatal degeneration via the transcriptional dysregulation of several genes, including genes that are involved in the calcium (Ca^2+^) signalosome. Recent research has shown that one of the major Ca^2+^ signaling pathways, store-operated Ca^2+^ entry (SOCE), is significantly elevated in HD. SOCE refers to Ca^2+^ flow into cells in response to the depletion of endoplasmic reticulum Ca^2+^ stores. The dysregulation of Ca^2+^ homeostasis is postulated to be a cause of HD progression because the SOCE pathway is indirectly and abnormally activated by mutant huntingtin (HTT) in γ-aminobutyric acid (GABA)ergic medium spiny neurons (MSNs) from the striatum in HD models before the first symptoms of the disease appear. The present review summarizes recent studies that revealed a relationship between HD pathology and elevations of SOCE in different models of HD, including YAC128 mice (a transgenic model of HD), cellular HD models, and induced pluripotent stem cell (iPSC)-based GABAergic medium spiny neurons (MSNs) that are obtained from adult HD patient fibroblasts. SOCE in MSNs was shown to be mediated by currents through at least two different channel groups, Ca^2+^ release-activated Ca^2+^ current (I_CRAC_) and store-operated Ca^2+^ current (I_SOC_), which are composed of stromal interaction molecule (STIM) proteins and Orai or transient receptor potential channel (TRPC) channels. Their role under physiological and pathological conditions in HD are discussed. The role of Huntingtin-associated protein 1 isoform A in elevations of SOCE in HD MSNs and potential compounds that may stabilize elevations of SOCE in HD are also summarized. Evidence is presented that shows that the dysregulation of molecular components of SOCE or pathways upstream of SOCE in HD MSN neurons is a hallmark of HD, and these changes could lead to HD pathology, making them potential therapeutic targets.

## Introduction

Calcium (Ca^2+^) ions are important universal second messengers that regulate numerous cellular processes. Ca^2+^ concentrations are strictly regulated by multiple Ca^2+^ channels, pumps, exchangers, and protein buffers. Under resting conditions, intracellular Ca^2+^ levels oscillate within the range of 100–300 nM. Upon cell activation, an increase in cytosolic Ca^2+^ concentrations in the range of 50–100 μM can occur in the form of microdomains by both the influx of Ca^2+^ ions through the plasma membrane (PM) or Ca^2+^ release from intracellular stores, such as the endoplasmic reticulum (ER) (Targos et al., 2005; Kiselyov et al., 2006; McCarron et al., 2006; Majewski and Kuznicki, 2015). Store-operated Ca^2+^ entry (SOCE) is the main Ca^2+^ entry pathway in non-excitable cells (Parekh and Putney, 2005; Prakriya and Lewis, 2015; Putney et al., 2017), which was characterized in detail in immune cells (Parekh and Penner, 1997; Feske et al., 2001, 2005; Feske, 2011). Initially, the SOCE process was known as capacitative Ca^2+^ entry (Putney, 1986). SOCE is described as Ca^2+^ flow into cells through PM Ca^2+^ channels in response to the depletion of ER Ca^2+^ stores. Ca^2+^ is released from the ER upon the activation of inositol-1,4,5-triphosphate receptors (IP3Rs) (Berridge, 2002, 2009; Bezprozvanny, 2005; Mikoshiba, 2007) and ryanodine receptors (RyRs) (Rossi and Sorrentino, 2002; Amador et al., 2013) and through passive leakage (van Coppenolle et al., 2004; Tu et al., 2006; Supnet and Bezprozvanny, 2011). The Ca^2+^ response via IP3Rs is known as IP3-induced Ca^2+^ release (IICR) (Miyazaki et al., 1993; Mikoshiba, 2007), whereas RyRs act according to Ca^2+^-induced Ca^2+^ release (CICR) (Berridge, 1998, 2002; Verkhratsky, 2005). Ca^2+^ release by IICR is often amplified by CICR (Berridge, 2002; Chen et al., 2011). RyRs are stimulated to transport Ca^2+^ into the cytoplasm by recognizing Ca^2+^ on its cytoplasmatic side, thus creating a positive feedback mechanism (Santulli and Marks, 2015). Ca^2+^ concentrations in the ER are detected by two stromal interaction molecule (STIM) isoforms, STIM1, and STIM2 (Liou et al., 2005; Roos et al., 2005; Zhang et al., 2005). They are localized in ER membranes and function as sensors of Ca^2+^ concentrations. Upon a decrease in Ca^2+^ in the ER, STIM proteins oligomerize, and migrate to ER-PM junctions where they interact with highly selective Ca^2+^ channels, Orai1-3, at the PM (Feske et al., 2006; Peinelt et al., 2006; Prakriya et al., 2006; Vig et al., 2006) and form large complexes that are visible as puncta under a microscope (Potier and Trebak, 2008; Klejman et al., 2009; Gruszczynska-Biegala and Kuznicki, 2013; Shim et al., 2015). This interaction causes the SOCE process, specifically Ca^2+^ flow from the extracellular space into the cytoplasm (Liou et al., 2005; Cahalan, 2009). To refill ER stores, the sarco-endoplasmic reticulum Ca^2+^ adenosine triphosphatase (SERCA) pump transports Ca^2+^ ions to the ER (Berridge, 2002; Periasamy and Kalyanasundaram, 2007; Brini et al., 2013a; Majewski and Kuznicki, 2015). SOCE is mediated by two different currents. One of them is Ca^2+^ release-activated Ca^2+^ current (I_CRAC_), which is highly selective for Ca^2+^, non-voltage activated, and inwardly rectifying (Hoth and Penner, 1993; Lewis and Cahalan, 1995). The second is store-operated Ca^2+^ current (I_SOC_), which is characterized by non-selective outward current with distinct biophysical features, including greater conductance than I_CRAC_ (Golovina et al., 2001; Trepakova et al., 2001; Strübing et al., 2003; Ma et al., 2015; Lopez et al., 2016, 2020). STIM-regulated Orai channels contribute to I_CRAC_-mediated SOCE, whereas STIM operated transient receptor potential channel 1 (TRPC1) activity mediates I_SOC_ (Majewski and Kuznicki, 2015; Moccia et al., 2015; Secondo et al., 2018).

Ca^2+^ signaling regulates multiple cellular processes (Brini et al., 2013b). Its dysregulation is the postulated underlying mechanism of many disorders, including neurodegenerative diseases, such as Alzheimer's disease, Parkinson's disease, amyotrophic lateral sclerosis (Pchitskaya et al., 2018; Secondo et al., 2018), and Huntington's disease (HD) (Raymond, 2017; Pchitskaya et al., 2018).

Huntington's disease is a progressive neurodegenerative disorder with autosomal-dominant heritability that is caused by CAG trinucleotide repeat expansion in the huntingtin gene (*HTT*) (MacDonald et al., 1993). The *HTT* gene encodes huntingtin protein (HTT), which is around 350 kDa in size and ubiquitously expressed in the cytoplasm of all cell types (MacDonald et al., 1993). Mutant HTT (mHTT) contains an expansion of polyglutamine residues (polyQ) in its amino-terminal part (Ross, 2002). Expansion longer than 35 repeats in mHTT results in a polyglutamine tract that leads to mHTT aggregation and earlier HD onset (DiFiglia et al., 1997; Krobitsch and Lindquist, 2000; Langbehn et al., 2004; Gusella and MacDonald, 2006). CAG repeats in mHTT between 40 and 60 cause the onset of HD at 30–50 years of age. The onset of HD before the age of 21 and CAG repeats over 60 are characteristic of the juvenile form of HD (Quigley, 2017), which resembles a neurodevelopmental disorder (Switońska et al., 2018; Wiatr et al., 2018). The most affected cells in HD are γ-aminobutyric acid (GABA)ergic medium spiny neurons (MSNs) in the striatum (Vonsattel and DiFiglia, 1998; Zoghbi and Orr, 2000). The clinical manifestations of HD include chorea, dementia, and mood and cognitive impairments (Zoghbi and Orr, 2000; Bates et al., 2015). Juvenile HD patients often present with rigidity, dystonia, seizures, cognitive alterations, and psychiatric symptoms (Quigley, 2017). No effective treatments have been developed for HD. The available medications only delay progression of the disease or alleviate its symptoms. Therefore, identification of the molecular mechanisms of HD and potential treatment targets are needed. In HD, the cascade of neurodegenerative processes was suggested to be caused by disturbances in Ca^2+^ signaling (Pchitskaya et al., 2018) that appear to be related to HTT function. Although its function remains unclear, the highest levels of wildtype HTT are found in the brain. Mutant HTT forms aggregates in neuronal nuclei. mHTT inhibits the function of various proteins, such as key transcription factors and Ca^2+^ signaling components, thereby affecting Ca^2+^ homeostasis (Giacomello et al., 2013). Disturbances in the Ca^2+^ signalosome were found in HD models and post-mortem samples from HD patients (Hodges et al., 2006; Wu et al., 2011, 2016; Czeredys et al., 2013). Abnormal Ca^2+^ signaling is considered an early event in HD pathology (Pchitskaya et al., 2018), particularly the SOCE pathway that is elevated in HD (Wu et al., 2011, 2016, 2018; Czeredys et al., 2013).

The present review provides an overview of Ca^2+^ signaling via store-operated Ca^2+^ channels under physiological conditions in neurons and under pathological conditions, namely HD. The distribution of STIM, Orai, and TRPC proteins in neurons and their functions in both the maintenance of ER Ca^2+^ concentrations and the activity of SOCE are discussed. The dysregulation of neuronal SOCE channels (nSOCs) has been implicated in HD pathology, especially affecting dendritic spines. The role of SOCE and SOCE components in the formation and maturation of dendritic spines and their contribution to synaptic plasticity under physiological conditions are also discussed. Finally, recent findings are presented that support the role of molecular components of neuronal SOCE (nSOCE) and upstream pathways that regulate these processes in dendritic spine pathology in HD. Potential drug candidates are proposed that may restore normal SOCE in HD. An argument is made that nSOCE may be a novel therapeutic target for HD.

## Neuronal Ca^2+^ Signaling VIA Store-Operated Ca^2+^ Channels Under Physiological Conditions

The role of SOCE as the main Ca^2+^ entry pathway in non-excitable cells is well-established. An important role for SOCE in excitable cells in the central nervous system (CNS), such as cortical pyramidal neurons (Klejman et al., 2009; Gruszczynska-Biegala et al., 2011), dorsal root ganglion neurons (Gemes et al., 2011), striatal MSNs (Wu et al., 2011, 2016; Czeredys et al., 2017), hippocampal pyramidal neurons (Emptage et al., 2001; Baba et al., 2003; Samtleben et al., 2015), and cerebellar Purkinje neurons (Hartmann et al., 2014), has also been recently demonstrated. Moreover, growing evidence suggests the existence of synaptic nSOCE (Sun et al., 2014; Wu et al., 2016, 2018; Ryskamp D. et al., 2019). The presence of molecular components of SOCE in both the neuronal cell body (Klejman et al., 2009; Gruszczynska-Biegala and Kuznicki, 2013) and dendritic spines (Garcia-Alvarez et al., 2015; Korkotian et al., 2017) has been reported. Additionally, the ER was shown to extend over the entire neuron, including numerous spines that encrust the dendrites (Berridge, 2002). Both STIM isoforms, STIM1 and STIM2, are broadly but differentially expressed in the CNS. STIM1 is the predominant isoform in the cerebellum (Klejman et al., 2009; Skibinska-Kijek et al., 2009; Hartmann et al., 2014). STIM2 is most prominent in the hippocampus (Berna-Erro et al., 2009; Skibinska-Kijek et al., 2009) and cortex (Skibinska-Kijek et al., 2009; Kraft, 2015). In immune cells, STIM2.1 and STIM2.2 isoforms were identified, which have different properties in the regulation of SOCE. STIM2.1 is a negative regulator of SOCE, and STIM2.2 is the counterpart of STIM2 (Miederer et al., 2015). Both isoforms are equally expressed in MSNs (Czeredys et al., 2018), but their functions in the regulation of SOCE in neurons are unknown.

STIM1 has high affinity for Ca^2+^ and requires the higher depletion of Ca^2+^ from the ER during SOCE. STIM2 is characterized by slower aggregation kinetics, and the weaker depletion of ER Ca^2+^ stores is sufficient to activate it (Brandman et al., 2007; Parvez et al., 2008; Stathopulos et al., 2009; Gruszczynska-Biegala et al., 2011; Gruszczynska-Biegala and Kuznicki, 2013). STIM2 was found to stabilize basal Ca^2+^ levels in rat neuronal cortical cultures and cell lines of peripheral origin (Brandman et al., 2007; Gruszczynska-Biegala et al., 2011; Gruszczynska-Biegala and Kuznicki, 2013). The interaction between STIM2 and Orai1 is weak and causes poor channel activation, whereas STIM2 was found to trigger remodeling of the STIM1 C-terminus and facilitate STIM1/Orai1 coupling and the enhancement of Orai1 function when alterations of Ca^2+^ levels in the ER are not sufficiently low to initiate STIM1 responses (Subedi et al., 2018).

Three members of the Orai channel family have been identified: Orai1, Orai2, and Orai3. They all interact with STIM1 but have different inactivation and permeability properties (DeHaven et al., 2007; Lis et al., 2007). Orais are known as Ca^2+^ release-activated Ca^2+^ channels (CRACs) (Prakriya et al., 2006). Among them, Orai1 is a crucial pore subunit of the CRAC channel (Prakriya et al., 2006) that mediates higher currents compared with the other isoforms (Putney et al., 2017). The Orai1 channel functions as a hexamer (Hou et al., 2012; Cai et al., 2016; Yen et al., 2016). Orai1 is the dominant form in immune cells (Vaeth et al., 2017), but the highest expression levels of the Orai2 isoform were detected in the brain (Chen-Engerer et al., 2019). Orai3 is highly expressed in cancer cells (Vashisht et al., 2018). Apart from STIM-regulated Orai channels that contribute to Ca^2+^ release-activated Ca^2+^ current (I_CRAC_)-mediated SOCE, recent studies indicated that STIM-operated TRPC1 activity mediates I_SOC_ (Liu et al., 2003; Ambudkar et al., 2017). Among six known TRPC proteins, TRPC1, TRPC3, and TRPC4 are SOCE partners that are activated by the depletion of Ca^2+^ from stores, whereas the operation of TRPC5, TRPC6, and TRPC7 is store-independent (Ambudkar et al., 2007; Liu et al., 2007; Venkatachalam and Montell, 2007; Trebak et al., 2009; Putney and Tomita, 2012). Phospholipase C (PLC)-mediated phosphatidylinositol 4,5-bisphosphate (PI(_4,5_)P_2_) hydrolysis upon the (*S*)-3,5-dihydroxyphenylglycine (DHPG)-induced activation of metabotropic glutamate receptor 1/5 (mGluR1/5) may activate TRPCs in a store-independent manner with diacylglycerol (DAG) (Itsuki et al., 2014). TRPC1 is recruited to the PM by Rab4-dependent recycling, which is critical for TRPC1-STIM1 clustering within ER-PM junctions (de Souza et al., 2015). TRPC1 function depends on Orai1-mediated Ca^2+^ entry, which enables TRPC1 recruitment to the PM where it is subsequently activated by STIM1 (Ambudkar et al., 2017). TRPC proteins may form different heteromeric structures that are involved in SOCE (Goel et al., 2002; Hofmann et al., 2002; Strübing et al., 2003; Wu et al., 2004; Liu et al., 2005; Zagranichnaya et al., 2005; Sundivakkam et al., 2012). The most prevalent TRPC channels in the mammalian brain are TRPC1, TRPC4, and TRPC5, which are mainly expressed in the hippocampus, prefrontal cortex, and lateral septum (Fowler et al., 2007, 2012), whereas TRPC3 is highly expressed in cerebellar Purkinje cells (Hartmann et al., 2014).

In the CNS, Ca^2+^ influx is mainly controlled by voltage-gated Ca^2+^ channels (VGCCs) and ionotropic glutamate receptors, such as glutamate-sensitive *N*-methyl-D-aspartate receptors (NMDARs) and α-amino-3-hydroxy-5-methyl-4-isoxazolepropionic acid receptors (AMPARs) (Catterall, 2011; Paoletti et al., 2013; Henley and Wilkinson, 2016), whereas the SOCE pathway is activated under resting conditions to refill ER stores and governs spontaneous neurotransmitter release (Emptage et al., 2001; Baba et al., 2003; Gruszczynska-Biegala and Kuznicki, 2013; Moccia et al., 2015; Wegierski and Kuznicki, 2018). In Purkinje neurons, STIM1-mediated SOCE replenishes intracellular Ca^2+^ levels when VGCC activity is low (Hartmann et al., 2014). Recent data suggest that SOCE may play a role in synaptic plasticity in the CNS and participate in regulating spine morphogenesis, neuronal excitability, and gene expression (Moccia et al., 2015). The role of STIM2 in maintaining post-synaptic mushroom spines in hippocampal neurons was also reported (Sun et al., 2014; Kraft, 2015). Ca^2+^ influx via STIM2-mediated SOCE regulates Ca^2+^/calmodulin-dependent protein kinase II (CaMKII) and stabilizes mushroom spines that play a role in memory storage (Sun et al., 2014). STIM2 also regulates AMPAR trafficking and plasticity at hippocampal synapses (Yap et al., 2017). Garcia-Alvarez et al. showed that STIM2 localizes to dendritic spines, is enriched in the post-synaptic density, and is required for regular synaptic activity. STIM2 mediates cyclic adenosine monophosphate (cAMP)-dependent phosphorylation and trafficking of the GluA1 subunit of AMPARs to PM-ER junctions independently from SOCE (Garcia-Alvarez et al., 2015). The role of STIM2 in synaptic plasticity has been established (Moccia et al., 2015), but the roles of STIM1 and Orai1 in spine architecture are just emerging. Korkotian et al. recently reported the role of Orai1 in the formation, maturation, and plasticity of dendritic spines in developing hippocampal neurons (Korkotian et al., 2017). These authors found that upon store depletion, STIM2 co-localized with Orai1 in spines, and STIM1 was less mobile in moving to spines than STIM2. Furthermore, the presence of clusters of Orai1 correlated with the emergence of nascent spines on dendrites following the transient elevation of extracellular Ca^2+^ concentrations (Korkotian et al., 2017). The role of STIM1 in regulating the structural plasticity of L-type VGCC-dependent dendritic spines was reported (Dittmer et al., 2017). The NMDAR activation of L-type VGCCs was proposed to release Ca^2+^ from the ER, which consequently causes STIM1 aggregation, inhibits L-type VGCCs, enhances ER spine content, and stabilizes mushroom spines (Dittmer et al., 2017). These findings were consistent with previous studies by two independent research groups that found a direct interaction between STIM1 protein and L-type VGCCs and the role of STIM1 in inhibiting the depolarization-mediated opening of L-type VGCC depolarization (Park et al., 2010; Wang et al., 2010). The role of STIM proteins in synaptic plasticity is further supported by findings by our group that STIMs can interact with and induce Ca^2+^ influx through AMPARs (Gruszczynska-Biegala et al., 2016). Additionally, the role of STIM proteins as potential negative regulators of NMDA-stimulated Ca^2+^ signaling was shown in cortical neurons (Gruszczynska-Biegala et al., 2020). The overexpression of STIM1 in brain neurons in transgenic mice improved contextual learning and impaired long-term depression (Majewski et al., 2017). Furthermore, STIM1 protein is responsible for mGluR1-dependent synaptic transmission in cerebellar Purkinje neurons (Hartmann et al., 2014). Accumulating evidence indicates a role for STIM1 in neurogenesis (Somasundaram et al., 2014) and the proliferation and early differentiation of neural progenitor cells (NPCs) (Somasundaram et al., 2014; Gopurappilly et al., 2018). The knockdown of both STIM isoforms reduced SOCE and inhibited the entry of mouse embryonic stem cells into a neural lineage (Hao et al., 2014). In human embryonic stem cells (hESCs), SOCE but not VGCC-mediated Ca^2+^ entry was observed, thus confirming that SOCE could play an essential role in Ca^2+^ signaling that is important for the self-renewal and differentiation of hESCs (Huang et al., 2017) and supporting the role of molecular components of SOCE in brain development.

**Figure 1:**
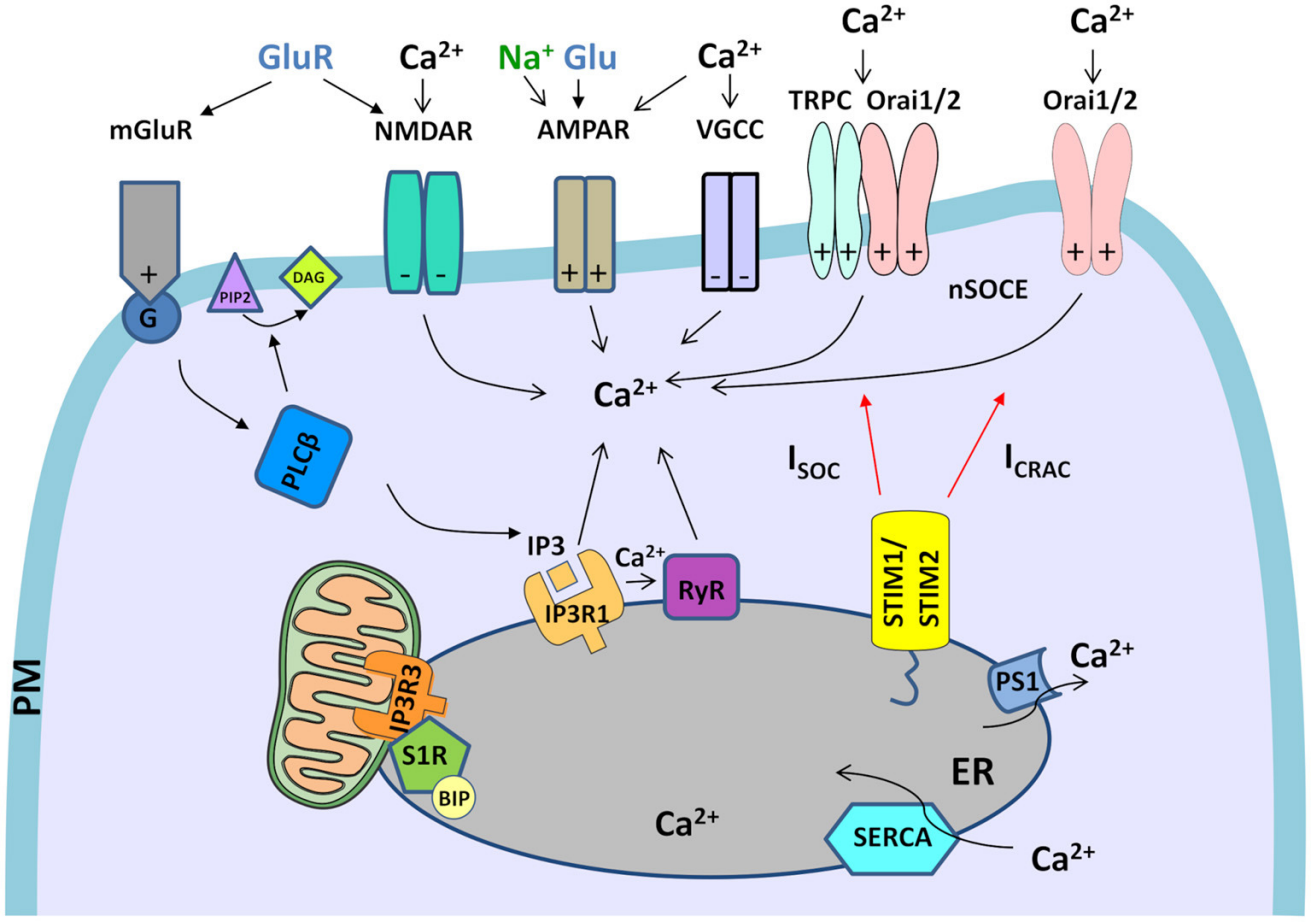
Neuronal Ca^2+^ signalosomes in physiology. Neuronal Ca^2+^ signaling is a complex process that involves Ca^2+^ influx from the extracellular space and Ca^2+^ release from ER, which contains the largest endogenous Ca^2+^ pool. Glutamate (Glu) is released from presynaptic neuronal terminals, which leads to stimulation of glutamate receptors (NMDARs, AMPARs, and mGluR1s), which are present in post-synaptic terminals of the excitatory synapses. Activation of Ca^2+^ efflux by NMDAR and VGCCs requires the excitatory post-synaptic potential that arises as a result of AMPAR activity that gates Na^+^ entry. VGCCs are activated only by depolarized membrane potential. In NMDAR activation excitatory post-synaptic potential removes the Mg^2+^ block from the channel what allows its opening in response to Glu stimulation and mediates Ca^2+^ influx. mGluR is activated by Glu and coupled to trimeric G-protein, which activates a membrane-associated enzyme, PLCβ, that mediates the hydrolysis of PI(_4,5_)P_2_ phospholipid to diacylglycerol (DAG) and signaling molecule, IP3. Then, IP3 interacts with IP3R receptors in the ER what causes the release of Ca^2+^ from ER stores. Further, Ca^2+^ release from the ER is exaggerated by RyR receptors in the ER, which are activated by Ca^2+^ release via IP3Rs through a mechanism called Ca^2+^-induced Ca^2+^ release (CICR). The activation of RyRs via NMDARs or VGCCs in the plasma membrane (PM) could also be involved in CICR (not shown in the figure). Upon depletion of ER Ca^2+^ stores, Ca^2+^ influx from extracellular space is caused by neuronal store-operated Ca^2+^ entry (nSOCE), which is mediated by the interaction between the ER Ca^2+^-sensors, Stim1 and Stim2, and the Ca^2+^-permeable channels, consisting of Orai1 and Orai2 and/or Orai and TRPC channels. These interactions mediate two types of currents, I_CRAC_ and I_SOC_. To refill ER Ca^2+^ stores SERCA pumps Ca^2+^ from the cytosol to the ER, while presenilin 1 (PS1) causes Ca^2+^ leak from the ER. Crosstalk between STIM proteins and receptors/channels in PM that was observed in neurons is marked on each receptor/channel as “+”–positive regulator/effector of STIM or “–“–negative effector of STIM. The only mGluR is the regulator of STIM. Within mitochondria-associated membranes (MAMs), the S1R receptor interacts with binding immunoglobulin protein (BIP), thereby regulates lipid dynamics and chaperones IP3R3 to the MAMs and facilitates Ca^2+^ flux from the ER to mitochondria. Filled black and red arrows represent interaction mechanisms. Open black arrows represent Ca^2+^/Na^+^ flux. Parts of the Figures 1–3 were drawn by using pictures from Servier Medical Art (http://smart.servier.com/), licensed under a Creative Commons Attribution 3.0 Unported License (https://creativecommons.org/licenses/by/3.0/).

Neuronal Ca^2+^ signaling is a complex process that involves Ca^2+^ inflow from the extracellular space and Ca^2+^ discharge from ER. Apart from the main molecular components of SOCE and PM receptors, several other molecules regulate Ca^2+^ signaling in neurons (Figure 1). The list of Ca^2+^ signalosomes directly or indirectly regulating Ca^2+^ signaling in neurons and their main physiological function were listed in Table 1. One of them is HTT binding partner, Huntingtin-associated protein-1 (HAP1), which facilitates functional effects of HTT on IP3R1 in planar lipid bilayers (Tang et al., 2004). In neurons, both HTT and HAP1 are involved in cytoskeleton regulation (Ma et al., 2011) and intracellular trafficking (Caviston and Holzbaur, 2009; Wu and Zhou, 2009). Another Ca^2+^ signalosome that does not directly regulate Ca^2+^ signaling is calcyclin binding protein and Siah-1 interacting protein (CacyBP/SIP), which interacts with Ca^2+^ binding protein calcyclin (Filipek et al., 2002), and its role was found, among others, in the cytoskeleton regulation (Jurewicz et al., 2013). There are several important signaling molecules in the ER that include presenilin 1 (PS1), which is a Ca^2+^ leak channel in the ER (Tu et al., 2006), the sigma-1 receptor (S1R), which is an ER-resident transmembrane protein regulating ER Ca^2+^ homeostasis (Su et al., 2010), and binding immunoglobulin protein (BiP), a chaperone protein, which maintains high Ca^2+^ levels in the ER (Hendershot, 2004). S1R interacts with BiP and prolong Ca^2+^ signaling from ER into mitochondria by stabilizing IP3R3s at the mitochondria-associated membranes (MAMs) (Hayashi and Su, 2007). It also blocks the inhibitory actions of ankyrin on IP3R3 (Wu and Bowen, 2008). In MSNs, where IP3R1 is a predominant neuronal isoform (Czeredys et al., 2018) S1R resisting in MAMs and stabilizes IP3R3 (Ryskamp D. A. et al., 2019). The S1R directly binds to IP3R1 and leads to the stimulation of protein kinase C (PKC) activity and suppression of IP3 synthesis in hepatocytes (Abou-Lovergne et al., 2011).

**Table 1:** Ca^2+^ signalosome directly or indirectly regulating Ca^2+^ signaling in neurons.

**Ca^**2+**^ signalosome**	**Identity**	**Subcellular location**	**Physiological function**	**References**
**AMPAR**	Ionotropic receptor	PM	Glutamate receptor and cation channel; involved in synaptic plasticity; positive effector of STIM1 and STIM2	Gruszczynska-Biegala et al., 2016; Henley and Wilkinson, 2016
**BIP**	Chaperone protein	ER	Maintain high Ca^2+^ levels in the ER; interacts with S1R what stabilize IP3R3 in MAMs; senses stress and activate unfolded protein response	Hendershot, 2004; Hayashi and Su, 2007
**CacyBP/SIP**	Phosphatase	Cytosol/nucleus	Interact with Ca^2+^ binding protein calcyclin; a modulator of the cytoskeleton	Filipek et al., 2002; Jurewicz et al., 2013
**CaMKII**	Kinase	PSD (cytoskeleton)	Activated by Ca^2+^ influx via STIM2 stabilizes mushroom spines by regulation of PSD95 and Cdc42	Sun et al., 2014
**DAG**	Second messenger signaling lipid	PM	Activates TRPC in a store-independent manner	Itsuki et al., 2014
**HAP1**	Interacting protein	Cytosol	Facilitates effects of HTT on IP3R1; involved in intracellular trafficking	Tang et al., 2004; Wu and Zhou, 2009
**HTT**	Interacting protein	Cytosol/nucleus	Activates IP3R1; involved in intracellular trafficking	Tang et al., 2004; Caviston and Holzbaur, 2009
**IP3**	Second messenger signaling molecule	Cytosol	Activates IP3R1	Bezprozvanny, 2005; Mikoshiba, 2007
**IP3R**	Ca^2+^ channel	ER	Releases Ca^2+^ from the ER	Bezprozvanny, 2005; Mikoshiba, 2007
**mGluR1/5**	Metabotropic glutamate receptor	PM	Glutamate receptor; modulates activation of other receptors; induces a biochemical cascade of IP3 production; positive regulator of STIM1	Tang et al., 2005; Hartmann et al., 2014
**NMDAR**	Ionotropic receptor	PM	Glutamate receptor and cation channel; involved in synaptic plasticity; negative effector of STIM1 and STIM2	Paoletti et al., 2013; Gruszczynska-Biegala et al., 2020
**Orai**	Ca^2+^ channel	PM	Ca^2+^ channel; positive effector of STIM1	Feske et al., 2006; Peinelt et al., 2006; Prakriya et al., 2006
**PI(**_**4,5**_**)P**_**2**_	Phospholipid	PM	Substrate for hydrolysis of IP3 and DAG by PLC	Itsuki et al., 2014
**PLC**	Membrane-associated enzyme	PM	Mediates PI(_4,5_)P_2_ hydrolysis to DAG and IP3	Itsuki et al., 2014
**PS1**	Ca^2+^-leak channel	ER	Ca^2+^ leak channels in the ER	Tu et al., 2006
**RyR**	Ca^2+^ channel	ER	Releases Ca^2+^ from the ER	Rossi and Sorrentino, 2002; Amador et al., 2013
**SERCA**	Ca^2+^-ATPase	ER	Refill ER Ca^2+^ stores	Periasamy and Kalyanasundaram, 2007; Brini et al., 2013a
**S1R**	Chaperone protein	ER	In MAMs regulates lipid dynamics and Ca^2+^ flux from the ER to mitochondria	Hayashi and Su, 2003, 2007; Su et al., 2010; Ryskamp D. A. et al., 2019
**STIMs**	Ca^2+^ sensor	ER	Ca^2+^ sensors in the ER	Liou et al., 2005; Roos et al., 2005; Zhang et al., 2005
**TRPC**	Ca^2+^ channel	PM	Ca^2+^ channel; positive effector of STIM1	Liu et al., 2003; Ambudkar et al., 2017
**VGCC**	Ca^2+^ channel	PM	Ca^2+^ channel activated at depolarized membrane potential; causes excitation of neurons; negative effector of STIM1	Park et al., 2010; Wang et al., 2010; Catterall, 2011; Dittmer et al., 2017

Besides the molecules that regulate SOCE, these processes might be regulated by rearrangement in cytoskeleton proteins. The role of the cytoskeleton in SOCE regulation through the modulation of the interaction between their main molecular components was demonstrated by several groups (Smyth et al., 2007; Grigoriev et al., 2008; Vaca, 2010; Galán et al., 2011; Giurisato et al., 2014). Septins are one of the cytoskeletal components that have been shown to facilitate interactions between STIM1 and Orai1 proteins (Sharma et al., 2013). In *Drosophila* neurons it was shown that dSEPT7 prevents dOrai-mediated spontaneous Ca^2+^ entry. Lower dSEPT7 levels lead to Ca^2+^ store-independent constitutive opening of dOrai channels and higher cytosolic Ca^2+^ in resting neurons, while dSEPT7 overexpression resulted in lower SOCE (Deb et al., 2016). Furthermore, it was shown in *Drosophila* neurons that microtubule stabilization by expression of a dominant-negative variant of the microtubule-severing protein spastin reduces SOCE, and decreases ER Ca^2+^ content. These effects were restored upon the application of a drug that stabilized microtubules (Vajente et al., 2019).

## Dysregulation of SOCE Causes Synaptic Loss in HD Models

Elevations of SOCE have been reported in several models of HD, including YAC128 MSNs (Wu et al., 2011, 2016; Czeredys et al., 2017). In YAC128 MSNs, an increase in STIM2 expression was observed in both YAC128 MSN cultures and 12-month-old YAC128 mice compared with controls. Increases in STIM2 expression elevated synaptic nSOCE, which likely was involved in synaptic loss in MSNs (Wu et al., 2016), whereas STIM1 expression was unchanged in HD MSNs. Indeed the CRISPR-Cas9-mediated knockdown of STIM2 caused the stabilization of dendritic spines in YAC128 MSNs. A key role for TRPC1 channels in supporting the SOC pathway was also shown in an HD model. The RNA interference (RNAi)-mediated knockdown of TRPC1 in YAC128 mouse MSNs exerted a significant protective effect against glutamate-induced apoptosis (Wu et al., 2011). Patch-clamp experiments in SK-N-SH cells transfected with full-length mutant HTT with 138Q (HTT-138Q) revealed that the current-voltage relationship of I_SOC_ currents was consistent with the involvement of TRP channels, and TRPC1 knockdown significantly reduced the magnitude of I_SOC_ currents in these cells (Wu et al., 2011). Additionally, crossing YAC128 mice with TRPC1 knockout mice improved motor performance and rescued MSN spines *in vitro* and *in vivo* (Wu et al., 2018). The RNAi-mediated knockdown of channels that are involved in SOCE other than TRPC1, such as TRPC6, Orai1, and Orai2, and knockout of the ER Ca^2+^ sensor STIM2 (Wu et al., 2016, 2018) resulted in the stabilization of spines and suppressed abnormal nSOCE in YAC128 MSNs. These results indicate that the molecular composition of the SOCE pathway in HD is complex and suggest the involvement of other players beyond STIM2 and TRPC1.

Apart from YAC128 transgenic mice, elevations of SOCE have also been described in a cellular model of HD (Wu et al., 2011; Vigont et al., 2014, 2015). In human neuroblastoma cells, expression of the N-terminal fragment of mHTT (exon-1 HTT with 138Q) increased STIM1-mediated SOCE (Vigont et al., 2014). Similar findings were reported in mouse neuroblastoma cells and primary cultures of mouse MSNs upon the lentiviral expression of N-terminal mHTT. Elevations of SOCE through actions of the sensor STIM1 and both TRPC1 and Orai1 subunits that comprise a heteromeric channel were found in these *in vitro* cultures (Vigont et al., 2015). Recent studies found that iPSC-based GABAergic MSNs from HD patient fibroblasts, which recapitulate some aspects of HD pathology, were characterized by elevations of SOC currents *in vitro* (Nekrasov et al., 2016). The SOCE of iPSC-based GABAergic HD MSNs was shown to be mediated by currents through at least two different channel groups, I_CRAC_ and I_SOC_. These currents were upregulated compared with wildtype iPSC-based GABAergic MSNs. Thapsigargin-induced intracellular Ca^2+^ store depletion in iPSC-based GABAergic MSNs resulted in the simultaneous activation of both I_CRAC_ and I_SOC_ (Vigont et al., 2018).

## Ca^2+^ Signaling Pathways That May Affect Soce and Cause Synaptic Loss in HD

Previous studies demonstrated that mHTT affects Ca^2+^ signaling in MSNs by increasing the sensitivity of IP3R1 to IP3 (Tang et al., 2003), facilitating Ca^2+^ release from internal Ca^2+^ stores (Ca^2+^ leakage) through RyRs (Suzuki et al., 2012), stimulating the activity of NR1/NR2B NMDARs (Chen et al., 1999; Sun et al., 2001; Zeron et al., 2002, 2004; Tang et al., 2005; Fan et al., 2007; Milnerwood and Raymond, 2007; Zhang et al., 2008), and disturbing mitochondrial Ca^2+^ handling (Panov et al., 2002; Choo et al., 2004; Oliveira et al., 2007). The increase in SOC pathway activity in HD may be caused directly by an increase in STIM2 expression in the presence of mHTT (Wu et al., 2016) or may result from a compensatory response of cells to the destabilization of Ca^2+^ signaling, such as an increase in the activity of IP3R1 by mHTT in YAC128 mice (Tang et al., 2003, 2004; Wu et al., 2016) or the modulation of SOC channels by receptors in the ER, such as S1Rs (Su et al., 2010).

A physiological mechanism that is responsible for activating SOCE results from the stimulation of G-protein-coupled receptors that are associated with the IP3 and PLC cascade, resulting in the release of Ca^2+^ from the ER via the IP3Rs (Streb et al., 1983). IP3R1 signaling is one of the pathways that act upstream of SOCE. Upon the release of Ca^2+^ from the ER via IP3R1, STIMs activate channels in the PM and cause Ca^2+^ influx to the cytosol. IP3R1 is abnormally activated in HD models that dysregulate ER Ca^2+^ dynamics. IP3R1 is the main IP3R isoform that is expressed in the striatum (Czeredys et al., 2018), and its activity is elevated in YAC128 MSNs (Wu et al., 2016). In MSNs, the overexpression of HTT-138Q activates IP3R1 more strongly compared with HTT that contains only 82Q repeats (Tang et al., 2003). Wu et al. reported that mHTT caused supranormal IP3R1 activity, which reduced ER Ca^2+^ levels (Wu et al., 2016). The depletion of ER Ca^2+^ leads to an increase in the activation of nSOCE in YAC128 MSN spines. Wu et al. showed that the inhibition of IP3R1 expression attenuated abnormal nSOCE and rescued spine loss in YAC128 MSNs (Wu et al., 2016), supporting the role of IP3R1 in neurite development (Fiedler and Nathanson, 2011). To inhibit IP3R1 activity, antisense oligonucleotides were applied, which resulted in the normalization of nSOCE and rescued spine loss in YAC128 MSNs (Wu et al., 2016). In summary, the role of IP3R1 that acts upstream of SOCE was established in synaptic loss in HD, in addition to known components of I_SOC_, including STIM2 and TRPC1 (Wu et al., 2016).

In our previous studies, we observed several abnormalities of Ca^2+^ signaling in YAC128 mice, such as the upregulation of some members of Ca^2+^ signalosomes in the striatum, including HAP1 and CacyBP/SIP (Czeredys et al., 2013). Their role has been recently established in neurodegenerative or neurodevelopmental diseases (Wasik et al., 2013; Xiang et al., 2017; Wang et al., 2019; Bohush and Filipek, 2020; Liu et al., 2020). Our group also observed the elevation of SOCE and an increase in IP3R1 activity in YAC128 MSNs (Czeredys et al., 2017). Our recent research focused on regulatory mechanisms of elevations of SOCE in MSNs from YAC128 mice (Czeredys et al., 2018). We found that the HAP1A isoform was responsible for the increase in SOC channel activity when it was overexpressed in MSNs from YAC128 mice. The HAP1B isoform differs from HAP1A in its C-terminal part and cannot interact with IP3R1 (Tang et al., 2003). HAP1B did not affect SOCE (Czeredys et al., 2018). We also observed a decrease in SOC channel activity when HAP1 protein was silenced. Upon HAP1A overexpression, an increase in IP3R1 activity and a decrease in the ionomycin-sensitive ER Ca^2+^ pool were observed. When the IP3R1 inhibitor IP3 sponge (Uchiyama et al., 2002) was applied via lentiviruses in YAC128 MSN cultures that overexpressed HAP1A, the increase in the release of Ca^2+^ from the ER and consequently the elevation of SOCE were both restored to wildtype levels. These experiments revealed that HAP1A elevates SOCE in the HD model through its interaction with mHTT and facilitation of the effects of mHTT on IP3R1 (Tang et al., 2003, 2004). To unravel the role of HAP1A in SOCE dysregulation in HD, we also used a cellular model of HD, SK-N-SH cells that express HTT138Q. We found that HAP1A overexpression constitutively activated SOC channels and upregulated STIM2 protein (Czeredys et al., 2018). Therefore, STIM2 may also be involved in the activation of DHPG-induced SOCE in YAC128 MSN cultures that overexpress HAP1A. The upregulation of STIM2 and hyperactivity of IP3R1 may underlie the constitutive activity of SOC channels in SK-N-SH-HTT-138Q-overexpressing HAP1A. Overall, our data indicate that HAP1A causes the aberrant activation of SOC channels in HD models. Thus, HAP1A appears to be an important player in HD pathology. Published data indicate that HAP1A dysregulates IP3R1 and upregulates STIM2. Therefore, we predict that elevations of SOCE in HD models may also underlie synaptic loss and neurodegeneration in HD.

In addition to IP3R1 signaling, which contributes to elevations of SOCE in HD models, another mechanism that involves S1Rs was recently reported (Ryskamp et al., 2017). The striatal upregulation of S1R was detected in aged YAC128 transgenic mice and HD patients, which caused ER Ca^2+^ dysregulation by IP3R1 modulation, an increase in SOCE, and abnormal dendritic spine morphology (Ryskamp et al., 2017). It was previously reported that upon ER Ca^2+^ depletion or ER stress or at high concentrations of S1R agonists, S1R is released from BIP, dislocates beyond the MAM domain (Su et al., 2010), and modulates additional targets, including IP3R1, STIM1, and ion channels and receptors on the PM (Kourrich et al., 2012; Ryskamp D. A. et al., 2019). The S1R was also reported to have functions in neuromodulation (Maurice et al., 2006) and neuroplasticity (Takebayashi et al., 2004; Tsai et al., 2009; Kourrich et al., 2012). The CRISPR/Cas9-mediated deletion of S1Rs resulted in MSN spine loss in wildtype corticostriatal co-cultures, thus indicating an essential role for S1Rs in the development or maintenance of synaptic connections between cortical and striatal neurons (Ryskamp et al., 2017).

Concentrations of Ca^2+^ in the ER are a determinant of the magnitude of Ca^2+^ signaling through IP3R and RyR channels. In HD models, the dysregulation of Ca^2+^ release from the ER by RyR signaling was also observed. Abnormal Ca^2+^ leakage by RyRs was detected in striatal and cortical neurons from an R6/2 mouse model of HD that overexpresses exon-1 mHTT (Suzuki et al., 2012). The involvement of RyRs in mHTT-induced neuronal death was also shown in R6/2 HD mice (Suzuki et al., 2012) and YAC128 mice (Chen et al., 2011). The association between SOCE and IP3R-gated Ca^2+^ stores has been thoroughly examined, but the role of RyR-gated stores in SOCE is less well-known. RyRs might play a role in synaptic plasticity (Baker et al., 2013; Johenning et al., 2015). A study that used an Orai1 dominant-negative mutant revealed the role of Orai1 in dendritic spine formation following the chemical induction of long-term potentiation (LTP) (Tshuva et al., 2017). This finding was attributable to the release of Ca^2+^ from RyR-associated ER stores. Spine formation in control neurons was reduced by the RyR antagonist dantrolene (Zucchi and Ronca-Testoni, 1997). Tshuva et al. postulated that Ca^2+^ stores are important for the formation of new dendritic spines. Under conditions of Orai1 deficiency, there is less Ca^2+^ in the stores, and Ca^2+^ release decreases, leading to decreases in LTP and spine formation (Tshuva et al., 2017).

The activation of SOCE by RyRs has been intensively studied in different cell types. RyR2-gated Ca^2+^ stores were shown to contribute to SOCE in pulmonary artery smooth muscle cells. However, depletion of RyR-sensitive Ca^2+^ store with caffeine was insufficient to activate Ca^2+^ entry but required a particular RyR conformation that was modify by ryanodine binding (Lin et al., 2016). Additionally, in primary human T cells, the RyR is related to CRAC machinery such that SOCE triggers RyR activation via a CICR mechanism following the attenuation of Ca^2+^ concentrations within the ER lumen in the proximity of STIM1, thereby facilitating SOCE by diminishing store-dependent CRAC inhibition (Thakur et al., 2012). Moreover, the RyR agonist 4-chloro-3-ethylphenol blocked Orai store-operated channels in rat L6 myoblasts and HEK293 cells (Zeng et al., 2014).

Apart from the effect of an increase in SOCE or its upstream pathways on the dysregulation of spines in HD, other pathways that involve disturbances in Ca^2+^ signaling have been proposed to be involved in HD pathology (Tang et al., 2005). One of them is the dysregulation of neurotransmitter release in synapses of HD neurons. A few recent studies found that mHTT can modulate N-type VGCCs (Ca_v_2.2), which are essential for presynaptic neurotransmitter release. In young BACHD mice that expressed full-length mHTT, an increase in striatal glutamate release was found, which was reduced to wildtype levels by Ca_v_2.2 inhibition. Ca_v_2.2 Ca^2+^ current density and PM expression also increased in these mice, which could be responsible for the elevation of glutamate release (Silva et al., 2017). The increases in synaptic vesicle release and elevations of Ca^2+^ influx at presynaptic terminals in primary cortical neurons were also found in a knock-in mouse model of HD (zQ175) (Chen et al., 2018). Moreover, under experimental conditions, the application of glutamate *in vitro* resulted in the apoptosis of YAC128 MSNs but not wildtype MSNs (Tang et al., 2005). Tang et al. found that abnormal glutamate release from corticostriatal projection neurons stimulated NR1/NR2B NMDARs and mGluR1/5 in YAC128 MSNs (Tang et al., 2005). Activation of NR1/NR2B NMDAR leads to Ca^2+^ influx and activation of mGluR5 receptors, leading to the production of IP3, which caused the subsequent activation of Ca^2+^ release via IP3R1. The stimulation of glutamate receptors results in supranormal Ca^2+^ responses in HD MSNs, which contributes to cytosolic Ca^2+^ overload and consequently exceeds mitochondrial Ca^2+^ storage capacity. This, in turn, guides the opening of mitochondrial permeability transition pores (MPTPs), release of cytochrome *c* into the cytosol, and activation of caspases 9 and 3 and apoptosis (Zeron et al., 2004; Tang et al., 2005). Although, the effect of the abnormal glutamate stimulation of NMDARs on dendritic spines of YAC128 MSNs have not been examined (Tang et al., 2005), these receptors may contribute to dendritic spine pathology in HD. Accumulating evidence suggests crosstalk between NMDARs and STIMs (Dittmer et al., 2017; Gruszczynska-Biegala et al., 2020). STIM1 was shown to regulate the structural plasticity of L-type VGCC-dependent dendritic spines. The NMDAR activation of L-type Ca^2+^ channels triggers Ca^2+^ release from the ER, which then regulates STIM1 aggregation, downregulates L-type channels, and enhances ER spine content and the stabilization of mushroom spines (Dittmer et al., 2017). The activation of NMDARs in pyramidal neurons causes the recruitment of IP3 through interactions with IP3R, leading to Ca^2+^ release from ER stores and SOCE stimulation. SOCE inhibitors reduced NMDA-dependent Ca^2+^ influx and synaptic plasticity in the hippocampus (Baba et al., 2003).

A few recent studies reported the role of AMPARs in HD pathology. In the striatum in HTTQ111/+ HD knock-in mice, synaptic dysfunction and disturbances in AMPAR-mediated signaling were observed. In the striatum in these mice, a decrease in synapse density, an increase in post-synaptic density thickness, and an increase in synaptic cleft width were observed. Acute slice electrophysiology showed alterations of spontaneous AMPAR-mediated post-synaptic currents, an increase in evoked NMDAR-mediated excitatory post-synaptic currents, and elevations of extrasynaptic NMDAR currents (Kovalenko et al., 2018). Cognitive impairments in rodent models of HD are linked to the improper diffusion of AMPARs in dendritic spines of hippocampal neurons through the dysregulation of brain-derived neurotrophic factor (BDNF)-TrkB-CaMKII signaling (Zhang et al., 2018). Moreover, in YAC128 mice, the AMPAR-dependent formation of new synapses through BDNF signaling is also disturbed (Smith-Dijak et al., 2019). Ca^2+^ influx via STIM2-mediated SOCE regulates CaMKII and stabilizes mushroom spines (Sun et al., 2014), but we cannot exclude the possible effects of other players of SOCE on abnormal AMPAR diffusion in spines in HD that were shown to act through BDNF-TrkB-CaMKII signaling (Zhang et al., 2018). Recent studies in rat cortical pyramidal neurons suggested that Ca^2+^ influx through AMPARs is induced by STIMs, which might confirm the relationship between SOCE and AMPARs (Gruszczynska-Biegala et al., 2016). These authors found that AMPAR antagonists inhibited SOCE, SOCE inhibitors decreased AMPA-induced Ca^2+^ influx, and both STIM1 and STIM2 proteins cooperated with GluA1 and GluA2 subunits of AMPARs (Gruszczynska-Biegala et al., 2016). However, other studies showed that STIM2 protein can interact with AMPARs in a SOCE-independent manner (Garcia-Alvarez et al., 2015). It was found that STIM2 regulated the phosphorylation of GluA1 at both Ser845 and Ser831, which promoted the cAMP/protein kinase A (PKA)-dependent surface delivery of GluA1 via exocytosis and endocytosis (Garcia-Alvarez et al., 2015).

## Potential Therapeutic Strategies to Stabilize SOCE in HD

In the striatum in HD transgenic mice, elevations of synaptic nSOCE were suggested to underlie post-synaptic dendritic spines loss in MSNs in aged corticostriatal co-cultures that were established from YAC128 mice (Wu et al., 2016). Several studies postulated that early neuropathological features of HD include perturbances of corticostriatal synaptic function and connectivity (Milnerwood and Raymond, 2007, 2010; Miller and Bezprozvanny, 2010; Orth et al., 2010; Murmu et al., 2013) and might lead to the accelerated neurodegeneration of MSNs in the striatum (Myers et al., 1988; Vonsattel and DiFiglia, 1998). Disturbances in the stability of synaptic spines have been suggested to underlie the development of HD symptoms (Bezprozvanny and Hiesinger, 2013; Murmu et al., 2013). The pharmacological inhibition of nSOCE that is abnormally elevated in HD could be a potential way to block neurodegeneration. A few SOCE inhibitors have been tested in HD models to date. One of these inhibitors is 6-amino-4-(4-phenoxyphenethyl-amino)quinazoline (EVP4593), which is a specific inhibitor of nSOC entry (Wu et al., 2011). A screen of a library of quinazoline-derived compounds was performed in HD flies that developed a progressive motor phenotype upon the induction of HTT-128Q transgene expression in the nervous system. In these flies, the HD phenotype was quantified using an automated climbing assay that automatically monitors motor functions. The application of EVP4593 normalized motor behavior in this fly model of HD and exerted neuroprotective effects in a glutamate toxicity assay in YAC128 MSN cultures, whereas its inactive analog EVP14808 failed to inhibit the SOC pathway in HD models (Wu et al., 2011). Further research revealed that EVP4593 reduces synaptic nSOCE and recovers abnormal spines in YAC128 MSNs. The intraventricular administration of EVP4593 in YAC128 mice *in vivo* rescued age-dependent striatal spine loss (Wu et al., 2016). Additionally, EVP4593 reduced SOCE to normal levels in MSNs that expressed exon-1 HTT with 138Q (Vigont et al., 2015). This compound also potently stabilized SOC entry in HD iPSC-based GABAergic MSNs (Nekrasov et al., 2016), thus confirming its specificity in neurons that were reprogrammed from fibroblasts from HD patients. The molecular target of EVP4593 is still unknown, but recent data showed that EVP4593 equally affected different SOC channels in HD iPSC-based GABAergic MSNs, suggesting that this compound could target SOCE regulatory proteins that are involved in both I_CRAC_ and I_SOC_ (e.g., STIM proteins) (Vigont et al., 2018). Huntington's disease iPSC-based GABAergic MSNs that were characterized by both excessive SOCE and progressive HD pathology (Nekrasov et al., 2016) were recently proposed to serve as a platform for personal drug screening in HD (Bezprozvanny and Kiselev, 2017; Vigont et al., 2018).

The stabilization of SOCE by tetrahydrocarbazoles was reported by our group in MSNs from YAC128 mice (Czeredys et al., 2017). We previously showed that these compounds decreased carbachol-induced Ca^2+^ efflux from the ER in HEK293-PS1-M146L cells, a cellular model of familial Alzheimer's disease (Honarnejad et al., 2014). Among seven selected tetrahydrocarbazoles, the most active compound in the HD model was 6-bromo-*N*-(2-phenylethyl)-2,3,4,9-tetrahydro-1*H*-carbazol-1-aminehydrochloride (C_20_H_22_BrClN_2_). This compound was able to restore disturbances in Ca^2+^ homeostasis and stabilize both DHPG- and CPA-induced SOCE in YAC128 MSN cultures. C_20_H_22_BrClN_2_ was added just 5 min before the measurements, and we concluded that it acts through the post-translational modification of proteins that are involved in SOCE, stabilization of the cytoskeleton, or the inhibition of SOCE or ER Ca^2+^ release channels. However, the effect of this tetrahydrocarbazole on the increase in Ca^2+^ release from the ER was not statistically significant, in contrast to its effect on SOCE. It specifically attenuated SOCE in YAC128 MSNs and did not affect SOCE in wildtype MSNs. We also found beneficial effects of this tetrahydrocarbazole on the function of mitochondria from YAC128 MSNs (Czeredys et al., 2017), which was consistent with its protective effects in the Alzheimer's disease model (Honarnejad et al., 2014). Our recent studies showed that the SOCE stabilizer C_20_H_22_BrClN_2_ reversed the elevation of SOCE in YAC128 MSN cultures that overexpressed HAP1A (Czeredys et al., 2018). In conclusion, this tetrahydrocarbazole stabilized SOCE in MSNs from YAC128 mice and might be a promising compound for the treatment of HD.

Synaptic nSOCE is suggested to underlie spines loss in YAC128 MSNs. Therefore, compounds that can inhibit elevations of SOCE could be beneficial for synapse maintenance in HD and possibly attenuating HD pathology. The effects of EVP4593 have already been established in the restoration of spines in HD models (Wu et al., 2016), but the effects of tetrahydrocarbazoles require further studies. Potential drugs that may inhibit elevations of SOCE and consequently attenuate spine abnormalities in HD neurons are summarized in Table 2.

**Table 2:** Potential strategies to stabilize SOCE in HD models.

**Potential treatment**	**Target**	**Effect on pathological mechanism in HD**	**References**
**EVP4593**			
*In vitro* (30–300 nM)	nSOCE inhibitor	• Stabilizes SOCE in YAC128 MSNs cultures	Wu et al., 2011
*In vivo* delivery (0.25 mg/ml via osmotic minipumps; Alzet)		• Normalizes motor behavior in a fly model of HD	
		• Exerts neuroprotective effects in a glutamate toxicity assay in YAC128 MSN cultures	
		• Reduces SOCE in MSNs that express exon-1 HTT-138Q	Vigont et al., 2015
		• Stabilizes synaptic nSOC and rescues spine loss in YAC128 MSNs	Wu et al., 2016
		• Rescues age-dependent striatal spine loss in YAC128 mice *in vivo*	
		• Stabilizes elevated I_SOC_ and I_CRAC_ currents in HD iPSC-based GABAergic MSNs	Vigont et al., 2018
**C**_**20**_**H**_**22**_**BrClN**_**2**_ (10 μM)			
	SOCE stabilizer	• Stabilizes both DHPG- and CPA-induced SOCE in YAC128 MSN cultures	Czeredys et al., 2017
		• Increases mitochondrial membrane potential in YAC128 MSNs	
		• Reverses the elevation of SOCE in YAC128 MSN cultures that overexpress HAP1A	Czeredys et al., 2018

## Potential Therapeutic Strategies to Stabilize Ca^2+^ Signaling in HD

**Figure 2:**
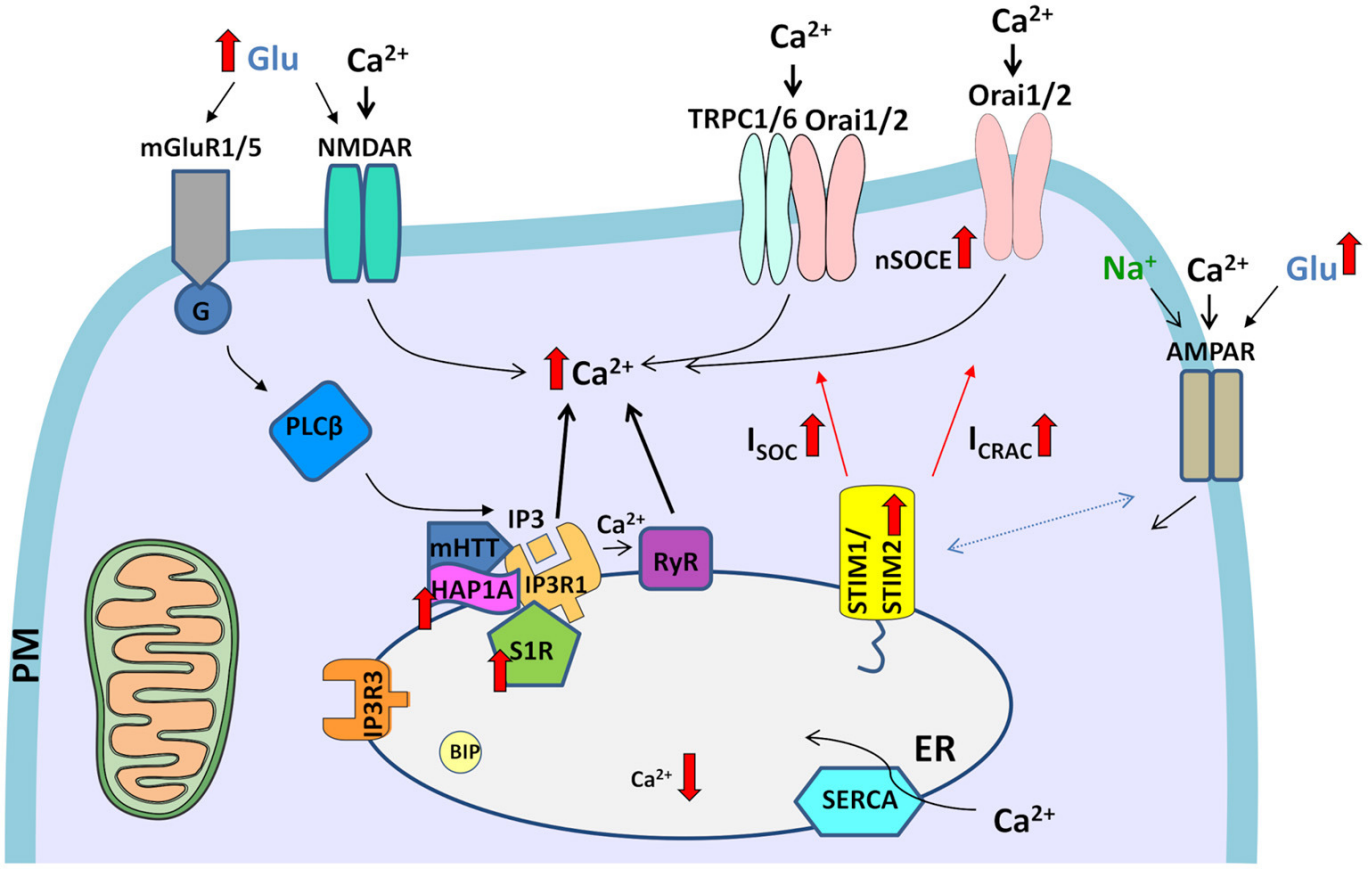
Dysregulation of Ca^2+^ signaling pathways in HD neurons. The elevation of glutamate (Glu) release from presynaptic neuronal terminals leads to the excessive stimulation of glutamate receptors (e.g., NMDARs and mGluR1/5) in post-synaptic terminals. The overactivation of NMDARs results in excessive Ca^2+^ influx. In turn, mGluRs couple to G-protein, which activates PLCβ and induces the formation of IP3, which interacts with IP3Rs and causes the release of Ca^2+^ from ER stores. HAP1A interacts with mHTT and exaggerates the effect of mHTT on IP3R1, which leads to the greater sensitivity of IP3R1 to IP3 and an increase in the release of Ca^2+^ from the ER, resulting in the attenuation of ER Ca^2+^ levels. Additionally, Ca^2+^ release from the ER is exaggerated by RyRs, which are activated by abnormal Ca^2+^ release via IP3Rs. Activated S1Rs via several processes, such as ER stress, Ca^2+^ release from the ER, or ligands, dissociate from binding immunoglobulin protein (BIP), thereby disconnecting from IP3R3 in mitochondria-associated membranes. Subsequently, upregulated S1R interacts with IP3R1. Upon the abnormal depletion of ER Ca^2+^ stores, upregulated STIM2 senses ER Ca^2+^ content and activates SOC channels in the plasma membrane (PM), which mediate two currents, I_SOC_ and I_CRAC_. Elevations of neuronal SOCE (nSOCE) contribute to Ca^2+^ overload. The surface distribution of AMPARs is disturbed in HD neurons. Crosstalk that was observed between AMPARs and STIMs in wildtype neurons might contribute to abnormal Ca^2+^ influx through AMPARs in HD pathology in the presence of upregulated STIM2. Filled black and red arrows represent interaction mechanisms. Open black arrows represent Ca^2+^/Na^+^ flux. Thick red arrows represent an increase or decrease in expression/concentration. Dashed arrows represent suspected interactions.

There are several points of mHTT interference with MSN Ca^2+^ signaling that are upstream of the SOCE pathway (Figure 2), and their dysregulation may cause elevations of SOCE in HD models, such as IP3R1 (Wu et al., 2016; Czeredys et al., 2018) and S1R (Ryskamp et al., 2017) signaling. Therefore, inhibitors of these pathways may also be promising drug candidates in HD.

One such drug is pridopidine, which was postulated to be a “dopamine stabilizer.” It has been shown to improve motor symptoms in clinical trials of HD (Lundin et al., 2010; de Yebenes et al., 2011; Esmaeilzadeh et al., 2011; Kieburtz et al., 2013). In previous studies, pridopidine improved motor functions and prolonged the survival in R6/2 HD mice, and exerted neuroprotective effects in a mouse striatal knock-in (STHdh111/111) model of HD (Squitieri et al., 2015). Further research has shown that the pharmacological activation of S1Rs with pridopidine and the chemically similar S1R agonist R(+)-3-(3-hydroxyphenyl)-*N*-propylpiperidine (3-PPP) prevents MSN spine loss in aging YAC128 co-cultures through the normalization of IP3R1 hyperactivity, suppression of abnormal ER Ca^2+^ release, restoration of ER Ca^2+^ levels, and reduction of excessive nSOCE in spines (Ryskamp et al., 2017). Pridopidine normalized ER Ca^2+^ levels, but its actions were prevented by S1R deletion. To evaluate the long-term effects of pridopidine, the expression profiles of Ca^2+^ signaling genes were analyzed. Pridopidine elevated the striatal expression of proteins that regulate Ca^2+^, such as calbindin and homer1a, whereas their striatal expression decreased in aged Q175KI and YAC128 HD mouse models compared with wildtype mice. Both pridopidine and 3-PPP restored Ca^2+^ dysregulation and synaptic loss in corticostriatal co-cultures from YAC128 mice (Ryskamp et al., 2017). Moreover, another S1R agonist, PRE-084, exerted neuroprotective effects in PC6.3 cells that expressed N-terminal mHTT (Hyrskyluoto et al., 2013).

An RyR antagonist and the clinically relevant intracellular Ca^2+^ stabilizer dantrolene was shown to protect cultured YAC128 MSNs against glutamate-induced apoptosis. Feeding dantrolene to YAC128 mice significantly attenuated age-dependent motor deficiency and decreased both the death of NeuN-positive striatal neurons and nuclear aggregation of mHTT (Chen et al., 2011). These results indicate that inhibiting RyR-mediated CICR may be a possible therapeutic approach for the treatment of HD, and dantrolene could be a potent compound that can be applied as an HD medication. An increase in Ca^2+^ leakage was observed in striatal and cortical neurons from R6/2 HD mice that overexpressed exon-1 mHTT. The application of RyR inhibitors, such as dantrolene, ryanodine, 1,1′-diheptyl-4,4′-bipyridinium dibromide (DHBP), and ruthenium red, suppressed cell death in cortical neurons that overexpressed the N-terminal fragment of mHTT, whereas the addition of the potent RyR activator 4-chloro-m-cresol (CMC) increased mHTT toxicity. In contrast, 2-aminoethoxydiphenyl borate (2-APB), an inhibitor of IP3Rs, failed to protect these neurons (Suzuki et al., 2012). These results suggest that the inhibition of Ca^2+^ release from RyRs but not IP3Rs alleviates neuronal death that is induced by exon-1 mHTT. Additionally, intracellular Ca^2+^ imaging revealed that exon-1 mHTT caused excessive basal Ca^2+^ release (Ca^2+^ leakage) through RyRs, leading to the depletion of internal Ca^2+^ stores. Moreover, the expression of FK506-binding protein 12 (FKBP12), which interacts and stabilizes RyR1 by decreasing channel open permeability (Brillantes et al., 1994), suppressed both Ca^2+^ leakage and cell death. These results provide evidence of the role of RyRs in neuronal cell death and suggest that the stabilization of RyRs might be beneficial for the treatment of HD (Suzuki et al., 2012).

Apart from small-molecule compounds that have been proposed for the treatment of HD, antisense oligonucleotides (ASOs) may also target mHTT in HD patients (Skotte et al., 2014; van Roon-Mom et al., 2018; Barker et al., 2020). Antisense oligonucleotides are short, synthetic, single-stranded oligodeoxynucleotides that can alter mRNA expression through various mechanisms and modify protein expression (Rinaldi and Wood, 2018). In addition to the ability of ASOs to decrease mHTT levels, other strategies to normalize the dysregulation of Ca^2+^ signaling have been proposed. The knockdown of IP3R1 by ASOs (536178-2) stabilized the supranormal steady-state activity of IP3R1 in YAC128 MSNs in corticostriatal co-cultures (Wu et al., 2016). Abnormal IP3R1 activation is a major cause of elevations of ER Ca^2+^ leakage that consequently leads to abnormal nSOCE in HD MSNs. Treatment with ASOs against IP3R1 rescued YAC128 MSN spines, increasing their numbers to the same levels as in wildtype cultures (Wu et al., 2016). Therefore, the ASO-mediated suppression of IP3R1 expression was sufficient to prevent spine loss in YAC128 MSNs, presumably through the stabilization of Ca^2+^ levels in the ER and nSOCE in neuronal spines. Wu et al. proposed that ASOs may be a promising treatment for HD because they can modify IP3R1 levels (Wu et al., 2016).

Moreover, IP3R1 sponge that was delivered using a lentivirus system *in vitro* into YAC128 MSNs that overexpressed HAP1A restored abnormal SOCE. IP3R1 sponge, through its ability to inhibit IP3R1 activity, attenuated the effects of mHTT and HAP1A on this receptor, which led to the normalization of SOCE in an HD model (Czeredys et al., 2018). Mutant HTT was previously shown to specifically bind to the C-terminal cytosolic region of IP3R1 (a 122-amino acid-long IC10 fragment) (Tang et al., 2003). These findings led Tang et al. to propose a novel therapy for HD, who introduced IC10 peptide into HD MSNs *in trans* to disrupt the pathogenic association between IP3R1 and mHTT to normalize neuronal Ca^2+^ signaling and prevent the cell death of HD MSNs. Indeed, infection with lenti-GFP-IC10 virus stabilized Ca^2+^ signaling in YAC128 MSNs *in vitro* and protected these neurons from glutamate-induced apoptosis. Intrastriatal injections of AAV1-GFP-IC10 attenuated motor deficits and diminished MSN loss and shrinkage in YAC128 mice *in vivo* (Tang et al., 2009). These results demonstrated the importance of IP3R1 and mHTT in the pathogenesis of HD and suggested that IC10 peptide may be useful for HD treatment. Additionally, IC10 peptide was shown to reduce mHTT aggregation and nuclear accumulation, which may suggest a novel protective mechanism of IC10 that works jointly with its stabilizing effects on Ca^2+^ signaling (Tang et al., 2009). Additionally, the IP3R blocker 2-APB (Maruyama et al., 1997) protected YAC128 MSNs from glutamate-induced apoptosis (Tang et al., 2005). Furthermore, low-molecular-weight heparin sulfate Enoxaparin inhibited IP3R1 activity and protected YAC128 MSNs from glutamate-induced apoptosis (Tang et al., 2005).

In summary, potential strategies to stabilize elevations of Ca^2+^ signaling pathways in the ER might be beneficial for HD patients because they may prevent abnormal SOCE and dendritic spine abnormalities. Possible treatment options that target Ca^2+^ signaling receptors in the ER, which may restore spine pathology in HD, are summarized in Table 3.

**Table 3:** Potential strategies to stabilize Ca^2+^ signaling pathways in the ER that may prevent abnormal SOCE in HD models.

**Potential treatment**	**Target**	**Effect on pathological mechanism in HD**	**References**
**Pridopidine**			
*In vivo* (5 or 6 mg/kg, i.p.)	Dopamine stabilizer; S1R activator	• Improves motor functions and prolongs survival of R6/2 HD mice	Squitieri et al., 2015
*In vitro* (150 μM)		• Antiapoptotic effects in knock-in cellular HD model (STHdh111/111)	Squitieri et al., 2015
*In vitro* (100 nM or 1 μM)		• Activates S1Rs, which prevents MSN spine loss in YAC128 co-cultures; suppresses supranormal ER Ca^2+^ release; restores ER calcium levels; reduces excessive synaptic nSOC	Ryskamp et al., 2017
**3-PPP**			
*In vitro* (100 nM or 1 μM)	S1R agonist	• Activates S1Rs, which prevents MSN spine loss in YAC128 co-cultures; restores excessive synaptic nSOC	Ryskamp et al., 2017
**PRE-084**			
*In vitro* (0.3 μM)	S1R agonist	• Neuroprotective properties in PC6.3 cells that express N-terminal mHTT	Hyrskyluoto et al., 2013
**ASO 536178-2 (Isis Pharmaceuticals)**			
*In vitro* (500 nM)	Knockdown of IP3R1	• Stabilizes the supranormal steady-state activity of IP3R1 in YAC128 MSNs co-cultures • Prevents spine loss in YAC128 MSNs through the stabilization of Ca^2+^ levels in the ER and nSOC in neuronal spines	Wu et al., 2016
**IP3R1 sponge**			
*In vitro* p49-dTomato in pUltraChili	IP3R1 inhibitor	• Restores abnormal SOCE in YAC128 MSNs that overexpress HAP1A • Restores increase in Ca^2+^ release from the ER in YAC128 MSNs that overexpress HAP1A	Czeredys et al., 2018
**IC10 peptide**			
IC10 fragment of rat IP3R1 (122 aa length) *In vitro* pLenti-GFP-IC10 *In vivo* AAV1-GFP-IC10	IP3R1 inhibitor	• Disrupts the pathogenic association between IP3R1 and mHTT • Stabilizes Ca^2+^ signaling in cultured YAC128 MSNs and protects YAC128 MSNs from glutamate-induced apoptosis • Intrastriatal injections significantly alleviate motor deficits and reduce MSN loss and shrinkage in YAC128 mice *in vivo* • Reduces mHTT aggregation and nuclear accumulation	Tang et al., 2009
**2-APB**			
*In vitro* (400 μM)	IP3R1 inhibitor	• Protects YAC128 MSNs from glutamate-induced apoptosis	Tang et al., 2005
**Enoxaparin (Lovenox)**			
*In vitro* (200 μg/ml)	IP3R1 inhibitor	• Protects YAC128 MSNs from glutamate-induced apoptosis	Tang et al., 2005
**Dantrolene**			
*In vitro* (10–50 μM) *In vivo* feeding (5 mg/kg twice/week)	RyRs antagonist	• Protects cultured YAC128 MSNs from glutamate-induced apoptosis • Attenuates age-dependent motor deficits in YAC128 mice *in vivo* • Decreases death of NeuN-positive striatal neurons and nuclear aggregation of mHTT	Chen et al., 2011
*In vitro* (30 μM)		• Suppresses cell death in cortical neurons that are transfected with N-terminal mHTT-150Q	Suzuki et al., 2012
**Ryanodine**			
*In vitro* (10 μM)	RyR inhibitor *at nanomolar concentrations, ryanodine partially activates RyRs	• Suppresses cell death in cortical neurons that are transfected with N-terminal mHTT-150Q	Suzuki et al., 2012
**DHBP**			
*In vitro* (50 nM)	RyR inhibitor	• Alleviates Ca^2+^ leakage through RyRs in cortical neurons that are transfected with N-terminal mHTT-150Q • Suppresses cell death in cortical neurons that are transfected with N-terminal mHTT-150Q	Suzuki et al., 2012
**Ruthenium red**			
*In vitro* (10 nM)	RyR inhibitor	• Suppresses cell death in cortical neurons that are transfected with N-terminal mHTT-150Q	Suzuki et al., 2012

One of the causes of MSN degeneration in HD is dysregulation of the glutamate/Ca^2+^ signaling pathway. Antagonists of glutamate signaling pathways might have beneficial effects for the treatment of HD, especially because crosstalk between glutamate receptors and neuronal SOCE was recently reported (Serwach and Gruszczynska-Biegala, 2019). The non-competitive NMDAR antagonist memantine (Lipton, 2006) protected YAC128 MSNs from glutamate-induced cell death. Furthermore, another antagonist of glutamate signaling pathways, riluzole, which acts on the inhibition of glutamate release, was also protective against glutamate-induced cell death, although it was less potent than memantine (Wu et al., 2006). Memantine was also examined in a 2-year-long human clinical study of HD. Memantine retarded the progression of HD in patients in motor, functional, and behavioral tests (Beister et al., 2004). Neuroprotective effects of the NMDAR antagonist (+)MK801 and NR2B-specific NMDAR antagonist ifenprodil were also found in a glutamate-induced cell death assay in YAC128 MSNs (Tang et al., 2005). Tang et al. found that mGluR1/5 inhibition by a combination of MPEP and CPCCOEt reduced the glutamate-induced apoptosis of YAC128 MSNs to wildtype MSN levels, and a mixture of mGluR1/5 and NMDAR blockers [MPEP, CPCCOEt, and (+)MK801] abolished glutamate-dependent cell death in both wildtype and YAC128 MSNs (Tang et al., 2005).

Although crosstalk between AMPARs and SOCE has been shown (Serwach and Gruszczynska-Biegala, 2019), an inhibitor of AMPARs, the antidepressant tianeptine ([3-chloro-6-methyl-5,5-dioxo-6,11-dihydro-(c,f)-dibenzo-(1,2-thiazepine)-11-yl)amino]-7 heptanoic acid) restored synaptic deficits in HD models independently from the SOCE process. Tianeptine improved hippocampal synaptic and memory deficits and anxiety/depression-like behavior in HD mice, possibly through the modulation of BDNF signaling and AMPAR surface diffusion (Zhang et al., 2018). The AMPAR-dependent formation of new synapses in cortical neurons from HD mice through BDNF signaling was restored by pridopidine, a drug that enhances BDNF signaling through the stimulation of S1Rs, and the S1R agonist 3-PPP (Smith-Dijak et al., 2019). Potential strategies to stabilize glutamate receptors in the PM that might affect SOCE in HD models are presented in Table 4.

**Table 4:** Potential strategies to stabilize receptors in the plasma membrane that may affect nSOCE in HD models.

**Potential treatment**	**Target**	**Effect on pathological mechanism in HD**	**References**
**Memantine**			
*In vitro* (10 μM)	Non-competitive NMDAR antagonist	• Protects YAC128 MSNs from glutamate-induced cell death	Wu et al., 2006
Up to 30 mg/ day in HD patients		• Retards the progression of motor, functional, and behavioral deficits in HD patients	Beister et al., 2004
**Riluzole**			
*In vitro* (10 μM)	Inhibition of glutamate release	• Protects against glutamate-induced cell death in YAC128 MSNs	Wu et al., 2006
**(+)MK801** *In vitro* (10 μM)	NMDAR antagonist	• Neuroprotective against glutamate-induced cell death in YAC128 MSNs	Tang et al., 2005
**Ifenprodil**			
*In vitro* (20 μM)	NR2B-specific NMDAR antagonist	• Neuroprotective against glutamate-induced cell death in YAC128 MSNs	Tang et al., 2005
Mixture of 20 μM **MPEP** and 50 μM **CPCCOEt** *in vitro*	mGluR1/5 inhibitors	• Reduces the glutamate-induced apoptosis of YAC128 MSNs	Tang et al., 2005
**Tianeptine**			
25 mg/kg (i.p., daily for 8 weeks)	AMPAR inhibitor	• Improves hippocampal synaptic and memory deficits	Zhang et al., 2018
10 mg/kg (single injection)		• Reverses anxiety/depression-like behavior in HD mice by modulating BDNF signaling and AMPAR surface diffusion	
**Pridopidine**			
*In vitro* (1 μM)	S1R activator	• Restores disruption of homeostatic synaptic plasticity signaling in cortical pyramidal neurons in HD models • Restores AMPAR-dependent formation of new synapses through BDNF signaling in HD cortical neurons	Smith-Dijak et al., 2019
**3-PPP**			
*In vitro* (1 μM)	S1R agonist	• Restores disruption of homeostatic synaptic plasticity signaling in cortical neurons in HD models • Restores AMPAR-dependent formation of new synapses through BDNF signaling in HD cortical neurons	Smith-Dijak et al., 2019

**Figure 3:**
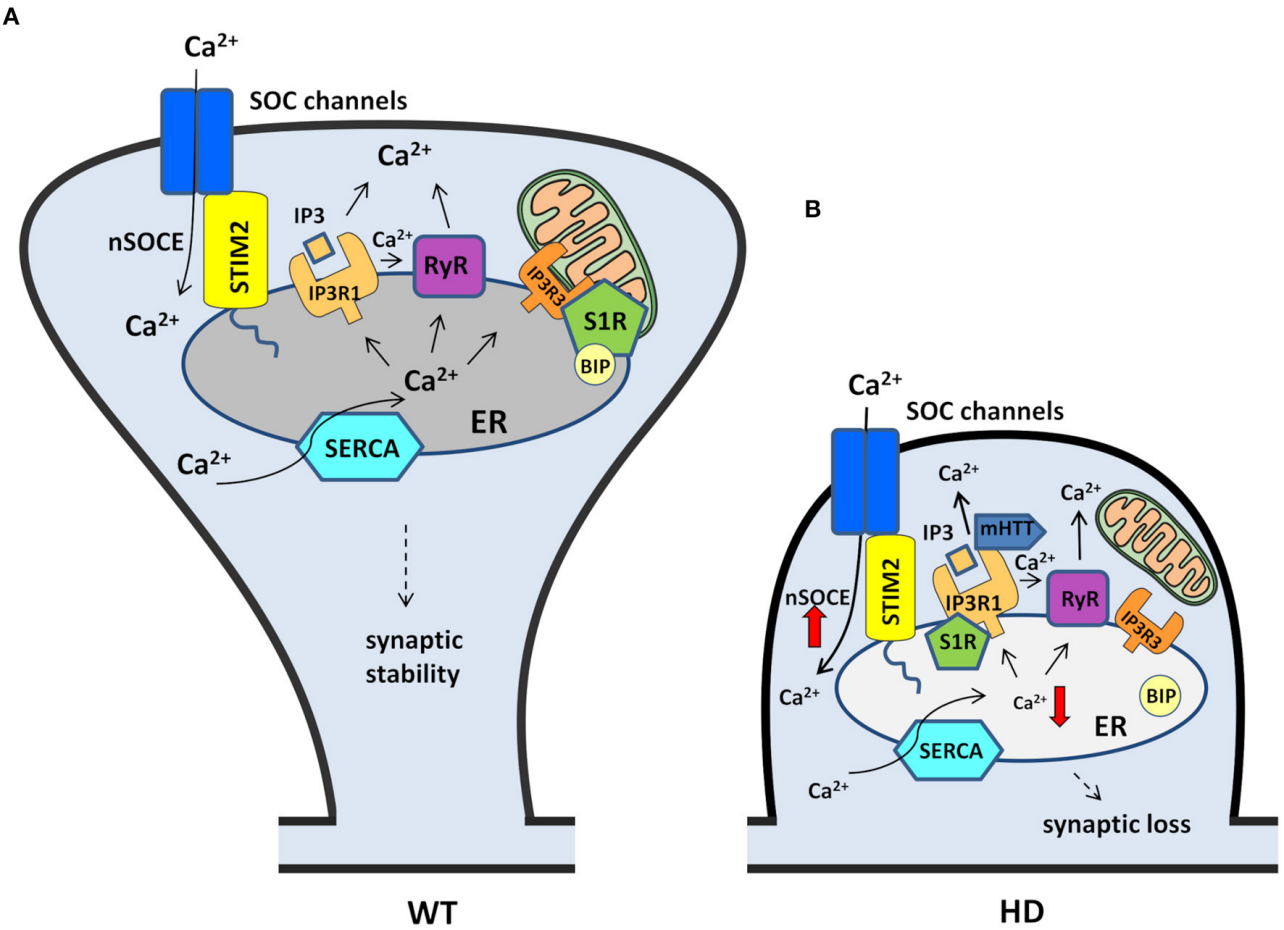
Contribution of nSOCE components and Ca^2+^ signaling pathways to the dysregulation of MSN spines in HD. **(A)** In wildtypes, MSN synaptic spine stability is maintained by the nSOCE pathway. nSOCE is gated by STIM2, which regulates ER Ca^2+^ levels by activating SOC channels in the plasma membrane, and SERCA pumps Ca^2+^ from the cytosol to the ER to refill ER Ca^2+^ stores. Ca^2+^ is released from the ER to the cytosol by IP3R1 and RyRs, and S1R facilitates Ca^2+^ flux from the ER to mitochondria via IP3R3. **(B)** In HD MSNs, supranormal synaptic nSOCE causes spine loss. mHTT sensitizes IP3R1 to IP3, causing excessive Ca^2+^ leakage from the ER. Ca^2+^ release from the ER is exaggerated by RyRs that are activated by abnormal Ca^2+^ release via IP3R1. Upon activation, S1Rs redistribute to the entire ER network where they interact with additional targets, including IP3R1, to affect their function. STIM2 upregulation compensates for the abnormal depletion of Ca^2+^ from the ER and causes the aberrant activation of nSOCE. Finally, supranormal nSOCE activates pathways and effectors that destabilize spines and lead to their loss in HD MSNs. Black arrows represent Ca^2+^ flux. The thick red arrows represent an increase or decrease in Ca^2+^ concentration.

The nSOC pathway plays an important role in supporting the stability of MSN spines under physiological conditions, and this process is disturbed in HD pathology. Both SOCE players and other Ca^2+^ signaling pathways that regulate nSOCE may contribute to the synaptic dysregulation of MSNs in HD (Figure 3). Substantial evidence suggests that the pharmacological inhibition of nSOCE, its upstream pathways, and other receptors that may affect the SOCE process may be beneficial for synapse maintenance in HD models. Although SOCE inhibition in the striatum might be protective in HD patients, the same compound might be detrimental in other brain regions that contain cortical and hippocampal pyramidal neurons where SOCE is unchanged (Secondo et al., 2018).

## Concluding Remarks

The dysregulation of Ca^2+^ signaling via store-operated Ca^2+^ channels is involved in the pathogenesis of HD. Abnormal nSOCE leads to synaptic dysregulation in HD MSNs. Several SOCE components have been identified in dendritic spine pathology. Among these, STIM2 and TRPC1 deserve special attention as potential HD treatment targets. Elevations of SOCE in HD models also result from abnormal Ca^2+^ signaling responses at the ER. The activity of two receptors in the ER, IP3R1, and S1R, is significantly elevated in HD models, which contributes to elevations of nSOCE and consequently MSN dendritic spine pathology. Additionally, the role of HAP1A protein should be emphasized because it can exaggerate the effect of mHTT on IP3R1, which leads to an increase in the release of Ca^2+^ from the ER and consequently elevations of SOCE in HD MSNs. One function of SOCE in neurons is to refill ER Ca^2+^ that is released during cell activation to initiate specific signaling pathways. However, SOCE also triggers various other signaling pathways. Evidence suggests crosstalk in neurons between glutamate receptors and SOCE components. Elevations of SOCE in HD might also be affected by glutamate receptors that are abnormally activated, which consequently causes MSN cell death. Under physiological conditions, SOCE components appear to be crucial in several aspects of neuronal development, including the proliferation and early differentiation of NPCs. Additionally, SOCE components play a role in synaptic formation and maturation, and they contribute to synaptic plasticity. Therefore, the dysregulation of SOCE appears to underlie dendritic spine abnormalities that have been identified in HD models. Recent findings that were reviewed herein support the role of molecular components of nSOCE and upstream pathways that regulate these processes in dendritic spine pathology in HD, and these components may be potential targets for HD treatment. Several drug candidates may restore abnormal SOCE (e.g., EVP4593 and tetrahydrocarbazoles) or other disturbances in Ca^2+^ signaling pathways, including IP3R1, RyR, and S1R. A few known compounds may prevent dendritic spine loss in HD by stabilizing abnormal nSOCE (e.g., EVP4593) and S1R activity (e.g., pridopidine and 3-PPP) or by attenuating supranormal IP3R1 activity (e.g., ASOs). The arguments that nSOC pathways could be novel therapeutic targets for HD treatment are convincing because elevations of SOCE were observed not only in transgenic and cellular HD models but also in iPSC-based GABAergic MSNs that were obtained from adult HD patient fibroblasts. However, such proposed drug candidates or other therapeutic strategies that may act on SOCE components in HD deserve further scrutiny to provide clear evidence that they can successfully and specifically target these proteins to improve or reverse the dysregulation of Ca^2+^ homeostasis and signaling without causing major side effects.

## Author Contributions

MC wrote the review and discussed literature data in this paper.

## Conflict of Interest

The author declares that the research was conducted in the absence of any commercial or financial relationships that could be construed as a potential conflict of interest.

## Abbreviations

AMPARs: α-amino-3-hydroxy-5-methyl-4-isoxazolepropionic acid receptors
2-APB: 2-aminoethoxydiphenyl borate
ASOs: antisense oligonucleotides
BDNF: brain-derived neurotrophic factor
BIP: binding immunoglobulin protein
CacyBP/SIP: calcyclin binding protein and Siah-1 interacting protein
CaMKII: Ca^2+^/calmodulin-dependent protein kinase II
cAMP: cyclic adenosine monophosphate
Ca_v_2.2: N-type voltage-gated calcium channel
Cdc42: cell division cycle 42
C_20_H_22_BrClN_2_: 6-bromo-*N*-(2-phenylethyl)-2,3,4,9-tetrahydro-1*H*-carbazol-1-aminehydrochloride
CICR: Ca^2+^-induced Ca^2+^ release
CMC: 4-chloro-m-cresol
CNS: central nervous system
CPA: cyclopiazonic acid
CRACs: Ca^2+^ release-activated Ca^2+^ channels
CRISPR-Cas9: clustered regularly interspaced short palindromic repeats/CRISPR-associated protein 9
DAG: diacylglycerol
DHBP: 1,1′-diheptyl-4,4′-bipyridinium dibromide
DHPG: (*S*)-3,5-dihydroxyphenylglycine
ER: endoplasmic reticulum
EVP4593: 6-amino-4-(4-phenoxyphenethyl-amino)quinazoline
FKBP12: FK506-binding protein 12
HAP1: huntingtin-associated protein 1
HD: Huntington's disease
hESCs: human embryonic stem cells
HTT: huntingtin
mHTT: mutant huntingtin
I_CRAC_: Ca^2+^ release-activated Ca^2+^ current
IICR: IP3-induced Ca^2+^ release
IP3: inositol trisphosphate
IP3Rs: inositol-1,4,5-triphosphate receptors
iPSCs: induced pluripotent stem cells
I_SOC_: store-operated Ca^2+^ current
LTP: long-term potentiation
MAM: mitochondria-associated membrane
mGluR1/5: metabotropic glutamate receptors1/5
MPTPs: mitochondrial permeability transition pores
MSNs: γ-aminobutyric acid (GABA)ergic medium spiny neurons
NMDARs: *N*-methyl-D-aspartate receptors
NPCs: neural progenitor cells
nSOCE: neuronal store-operated calcium entry
nSOCs: neuronal store-operated calcium channels
PKA: protein kinase A
PKC: protein kinase C
PI_(4,5)_P_(2)_: phosphatidylinositol 4,5-bisphosphate
PLC: phospholipase C
PM: plasma membrane
polyQ: polyglutamine residues
3-PPP: R(+)-3-(3-hydroxyphenyl)-*N*-propylpiperidine
PS1: presenilin 1
PSD95: PDZ-domain scaffold protein post-synaptic density 95
Rab4: Ras-related protein Rab4
RyRs: ryanodine receptors
SERCA: sarco-endoplasmic reticulum calcium-adenosine triphosphatase
SOCE: store-operated calcium entry
S1Rs: sigma-1 receptors
STIM: stromal interaction molecule
TrkB: tropomyosin receptor kinase B
TRPCs: transient receptor potential channels
VGCCs: voltage-gated calcium channels.
